# Clinical outcomes of chronic heart failure patients with unsuppressed sleep apnea by positive airway pressure therapy

**DOI:** 10.3389/fcvm.2023.1156353

**Published:** 2023-06-16

**Authors:** Ryo Naito, Takatoshi Kasai, Yasuhiro Tomita, Satoshi Kasagi, Koji Narui, Shin-Ichi Momomura

**Affiliations:** ^1^ Department of Cardiovascular Biology and Medicine, Juntendo University Graduate School of Medicine, Tokyo, Japan; ^2^ Cardiovascular Respiratory Sleep Medicine, Juntendo University Graduate School of Medicine, Tokyo, Japan; ^3^ Sleep Center, Toranomon Hospital, Tokyo, Japan; ^4^ Department of Medicine, Saitama Citizens Medical Center, Saitama, Japan

**Keywords:** heart failure, unsuppressed sleep apnea, continuous positive airway pressure therapy, death, clinical outcome

## Abstract

**Introduction:**

Heart failure (HF) is an advanced stage of cardiac disease and is associated with a high rate of mortality. Previous studies have shown that sleep apnea (SA) is associated with a poor prognosis in HF patients. Beneficial effects of PAP therapy that is effective on reducing SA on cardiovascular events, were not yet established. However, a large-scale clinical trial reported that patients with central SA (CSA) which was not effectively suppressed by continuous positive airway pressure (CPAP) revealed poor prognosis. We hypothesize that unsuppressed SA by CPAP is associated with negative consequences in patients with HF and SA, including either obstructive SA (OSA) or CSA.

**Methods:**

This was a retrospective observational study. Patients with stable HF, defined as left ventricular ejection fraction of ≤50%; New York Heart Association class ≥ II; and SA [apnea–hypopnea index (AHI) of ≥15/h on overnight polysomnography], treated with CPAP therapy for 1 month and performed sleep study with CPAP were enrolled. The patients were classified into two groups according to AHI on CPAP (suppressed group: residual AHI ≥ 15/h; and unsuppressed group: residual AHI < 15/h). The primary endpoint was a composite of all-cause death and hospitalization for HF.

**Results:**

Overall, data of 111 patients including 27 patients with unsuppressed SA, were analyzed. The cumulative event-free survival rates were lower in the unsuppressed group during a period of 36.6 months. A multivariate Cox proportional hazard model showed that the unsuppressed group was associated with an increased risk for clinical outcomes (hazard ratio 2.30, 95% confidence interval 1.21–4.38, *p* = 0.011).

**Conclusion:**

Our study suggested that in patients with HF and SA including either OSA or CSA, presence of unsuppressed SA even on CPAP was associated with worse prognosis as compared to those with suppressed SA by CPAP.

## Introduction

Heart failure (HF) is an advanced stage of cardiac disease and is associated with a high rate of mortality (1, 2). Detection of patients' backgrounds that contribute to an increased risk of mortality could aid in improving survival rates in HF patients. Several studies have reported that the presence of sleep apnea (SA) is associated with a poor prognosis in patients with HF (3). Continuous positive airway pressure (CPAP) therapy for SA, either obstructive or central SA (OSA and CSA, respectively) can improve cardiac function and probably clinical outcomes in those patients (4–7). However, a large-scale clinical trial reported patients with CSA which was not effectively suppressed by CPAP revealed poor prognosis (8). Only one study suggested such relationship between unsuppressed CSA and poor prognosis and there are no studies showing unsuppressed OSA by CPAP in patients with HF. We hypothesize that unsuppressed SA including either OSA or CSA by CPAP, is associated with negative consequences in patients with HF and SA. Therefore, we investigated that the prognosis of patients with unsuppressed SA by CPAP therapy in HD patients with SA including either OSA and CSA.

## Materials and methods

### Study population

Consecutive patients with HF and moderate to severe SA who were followed up at the cardiovascular center of Toranomon Hospital (Tokyo, Japan) between January 1, 2001, and March 1, 2005, were enrolled in the study.

The inclusion criteria were as follows: (1) HF with mildly reduced ejection fraction (HFmrEF) or those with reduced ejection fraction (HFrEF) on echocardiography within 1 month before the diagnostic sleep study and New York Heart Association (NYHA) class II or above; (2) stable clinical status, defined as no hospital admissions within 1 month before study enrollment and receiving optimal medical therapy for at least 1 month before study enrollment; (3) having undergone a diagnostic sleep study and received a diagnosis of moderate-to-severe SA, which was defined as an AHI ≥15; (4) receiving CPAP therapy and another sleep study with CPAP on 1 month after initiation. The exclusion criteria were as follows: (1) age below 20 or above 80 years, (2) presence of known untreated neoplasms, (3) history of stroke with neurologic deficits, and (4) history of severe chronic pulmonary diseases.

They were classified into two groups according to the AHI on CPAP (unsuppressed group, residual AHI ≥ 15/h; and suppressed group, residual AHI < 15/h). Informed consent was obtained from all the patients who participated in the study. The study was conducted in compliance with the Declaration of Helsinki and in accordance with the ethics policies of the institutions involved.

### Sleep study and CPAP

As sleep studies, overnight polysomnography was performed according to standard protocol and criteria (9). Electrocardiograms, electroencephalograms, electrooculograms, and electromyograms were performed, and thoracoabdominal motion was monitored using respiratory inductance plethysmography. Air flow was measured using an oronasal thermal airflow sensor and nasal pressure cannula or through nasal mask when sleep studies were done with CPAP, and oxyhemoglobin saturation (SO_2_) was monitored using oximetry. Respiratory events (i.e., apneas or hypopneas) were scored according to the American Academy of Sleep Medicine (AASM) scoring manual 2020 updates (10). Apnea with and without ribcage and/or abdominal movement was defined as obstructive and central apnea, respectively. Hypopnea was classified as obstructive if any of the following conditions existed: (1) paradoxical chest and abdominal movement during hypopnea events, (2) snoring during hypopnea events, or (3) flow limitation. Otherwise, the hypopneas were classified as central. We defined patients with predominantly CSA as having an AHI of ≥15/h, of which >50% were central events (5). All patients were offered CPAP therapy after the diagnosis of moderate to severe SA. The CPAP was titrated to determine the appropriate pressure level for each patient. In patients whose apnea or hypopnea could not effectively suppressed, the best pressure level which can well control obstructive respiratory events was determined. Patients were instructed to use the device with that pressure level while sleeping at home. One month after initiation of CPAP, patients underwent another polysomnography with CPAP and data from the polysomnography was used as baseline data on CPAP in the present study. Patients who stopped using CPAP after polysomnography with CPAP was determined as CPAP dropout cases.

### Other data

The following variables were obtained from the clinical chart at the time of polysomnography with CPAP: body mass index (BMI); blood pressure (BP); heart rate; LVEF on echocardiograms; plasma norepinephrine (PNE) and brain natriuretic peptide (BNP) levels; NYHA class; etiology of HF; the presence of atrial fibrillation (AF); and administered drugs. The frequency of death and hospitalization was also assessed. The end point was a composite of death and hospitalization. Follow-up ended on March, 2006, and the prognosis was assessed by analyzing the medical records of patients who died and of those who continued to be followed up at our hospital. Information about the circumstances and the date of death was obtained from the families of patients who died at home. The reasons for hospitalization or the causes of death were determined from the institutions to which the patients had been admitted.

### Statistical analysis

The data of all variables were presented as mean ± standard deviation or median and interquartile range. The baseline characteristics were compared using Student's *t*-test or the Mann–Whitney *U*-test for continuous variables, while the *χ*^2^ test or Fisher's exact test was used for categorical variables. Event-free survival between the groups was compared using the Kaplan–Meier estimate with the log-rank test, and hazard ratios (HRs) were calculated using the Cox proportional hazards model. Median follow-up time was calculated from the Kaplan-Meier product-limit estimates of the survival function. Univariate analysis was based on the proportional hazards model to determine the associations between prognosis and the following variables obtained at the time of polysomnography with CPAP: age, sex, BMI, AF etiology of HF, NYHA class, systolic or diastolic BP, heart rate, LVEF, BNP and PNE levels, sleep study data on CPAP such as total sleep time (TST), percentage of slow wave sleep and rapid eye movement (REM) sleep per TST, arousal index, percentage of time spent SO_2 _< 90% (%TST SO_2 _< 90%), lowest SO_2_, percentages of central AHI over total AHI (% central/total AHI), unsuppressed SA, and CPAP dropout cases. Variables with a *p* value below 0.1 in univariate analysis were included in multivariate analysis. Statistical significance was set at p < 0.05. All statistical analyses were performed using a statistical software package (SPSS, version 11.0 for Windows; SPSS Inc., Chicago, IL).

## Results

A total of 111 patients were enrolled in this study. Fifty patients had the primary outcomes (18 and 32 in the unsuppressed and the suppressed groups, respectively with the median follow-up period of 36.6 months. Characteristics of the patients are shown in Table 1. The unsuppressed group tended to be older and have lower diastolic BP, and have significantly higher PNE level than the suppressed group. A prevalence of atrial fibrillation was significantly higher in the unsuppressed group. However, there were no difference in other baseline characteristics between two groups. Polysomnographic data for diagnostic and on CPAP studies were shown in Table 2. On the diagnostic study, in unsuppressed group, total AHI was significantly greater with a higher %central/total AHI. Indeed, percentage of predominant CSA on diagnostic study was significantly high in unsuppressed group (77.8% in unsuppressed group vs. 29.8% in suppressed group, *p* < 0.001). In addition, percentage of slow wave sleep per TST was significantly lower in the unsuppressed group. In both groups, all parameters were significantly improved by CPAP except for the increased %central/total AHI and no changes in TST. Comparing on CPAP study data (i.e., baseline data for the survival analyses), AHI on CPAP was significantly higher with higher %central/toral AHI on CPAP and with more hypoxic burden. Following on CPAP study, 13 patients (15.5%) in the suppressed group and 7 patients (25.9%) in the unsuppressed group dropped out from their CPAP therapy (*p* = 0.345).

**Table 1:** Baseline characteristics of patients in the two groups.

	Suppressed (*n* = 84)	Unsuppressed (*n* = 27)	*p*
Age, years	60.9 ± 13.2	66.4 ± 11.6	0.054
Male sex, *n* (%)	80 (95.2)	24 (88.8)	0.358
BMI, kg/m^2^	26.4 ± 6.0	27.6 ± 6.1	0.397
Ischemic etiology, *n* (%)	24 (28.6)	8 (30.0)	0.916
AF, *n* (%)	28 (33.3)	17 (63.0)	0.012
NYHA class, *n* (%)			0.628
II	44 (52.4)	12 (44.4)	
III + IV	40 (47.6)	15 (55.6)	
Systolic BP, mmHg	127.5 ± 11.3	123.5 ± 11.3	0.160
Diastolic BP, mmHg	78.7 ± 8.3	74.3 ± 10.3	0.064
HR, /min	75.8 ± 10.2	73.8 ± 9.3	0.417
LVEF, %	41.8 ± 12.7	42.9 ± 11.3	0.724
BNP, pg/ml	134.0 (287.8)	153.0 (283.5)	0.477
PNE, pg/ml	528.0 ± 241.4	569.5 ± 171.1	0.040
Medications			
Beta-blockers, *n* (%)	48 (57.1)	18 (66.7)	0.381
ACE inhibitors/ARBs, *n* (%)	68 (81.0)	23 (85.2)	0.619
Aldosterone-blockers, *n* (%)	20 (23.8)	6 (22.2)	0.865
Diuretics, *n* (%)	68 (81.0)	20 (74.1)	0.443
Implantable cardiac device, *n* (%)			
Cardiac pacemaker, *n* (%)	9 (10.7)	2 (7.4)	0.248
Implantable cardioverter defibrillator, *n* (%)	4 (4.8)	0 (0)	0.617

Continuous data were shown as mean ± standard deviation or median (interquartile range).

ACE, angiotensin-converting enzyme; AF, atrial fibrillation; ARB, angiotensin II receptor blocker; BMI, body mass index; BNP, brain natriuretic peptide; BP, blood pressure; HR, heart rate; LVEF, left ventricular ejection fraction; NYHA, New York Heart Association; PNE, plasma norepinephrine.

**Table 2:** Polysomnographic findings.

	Suppressed (*N* = 84)			Unsuppressed (*N* = 27)		
	Diagnostic study	On CPAP study	*p* ^†^	Diagnostic study	On CPAP study	*p* ^†^
TST (min)	322.8 ± 79.1	336.8 ± 79.6	0.137	309.6 ± 94.4	341.6 ± 95.6	0.369
Total AHI no./h	44.2 ± 17.6	5.1 ± 3.6	<0.001	51.6 ± 14.2*	21.3 ± 7.5*	<0.001
%central/total AHI	21.6 (57.1)	31.9 (62.5)	0.001	81.6 (28.7)*	86.0 (38.6)*	0.023
%TST SO_2 _< 90% (%)	17.6 (46.4)	0.2 (1.2)	<0.001	19.1 (45.7)	2.1 (7.7)**	0.002
Lowest SO_2_%	75.3 ± 13.8	96.3 ± 1.6	<0.001	75.6 ± 13.0	84.9 ± 4.5*	0.002
Arousal index no./h	41.1 ± 19.6	15.8 ± 10.1	<0.001	43.6 ± 20.0	17.6 ± 8.4	<0.001
Sleep stage % of TST						
Slow wave sleep (%)	6.0 (12.5)	15.5 (13.1)	<0.001	0 (2.6)**	12.0 (21.5)	0.022
REM sleep (%)	9.9 ± 6.4	17.1 ± 7.1	<0.001	9.6 ± 5.8	18.3 ± 7.0	0.005

AHI, apnea–hypopnea index; CPAP, continuous positive airway pressure; REM, rapid eye movement; SO_2_, oxyhemoglobin saturation; TST, total sleep time.

Data were shown as mean ± standard deviation or median (interquartile range).

* *p* < 0.05 comparing with suppressed group.

** *p* < 0.01 comparing with suppressed group.

^†^ *p* for comparing between diagnostic and on CPAP studies.

**Figure 1:**
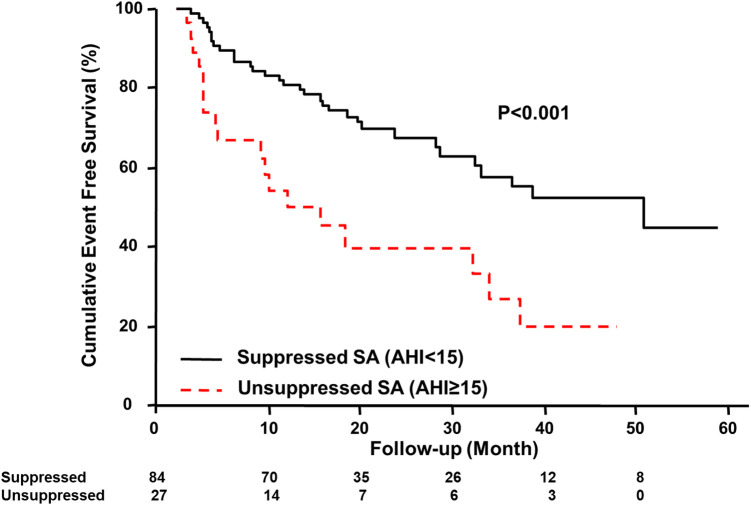
Kaplan–Meier estimation of event-free survival. Kaplan–Meier estimation of cumulative event-free survival for all-cause mortality and hospitalization for HF showing a significantly worse clinical outcome in the unsuppressed group. AHI, apnea-hypopnea index; SA, sleep apnea; HF, heart failure.

Kaplan-Meier estimation of cumulative event-free survival for all-cause mortality and hospitalization for HF revealed a significantly worse clinical outcome in the unsuppressed group compared to the suppressed group (log-rank test *p* < 0.001) (Figure 1). A multivariate Cox proportional hazard model showed that unsuppressed SA in addition to AF and CPAP dropout were associated with an increased risk for clinical outcomes (Table 3).

**Table 3:** Results of univariate and multivariate analysis assessing prognostic factors for clinical outcomes.

	Univariable			Multivariable		
	HR	95%CI	*P*	HR	95%CI	*P*
Age (1 y.o. increase)	1.03	1.01–1.06	0.014	1.01	0.98–1.04	0.483
Log-transformed BNP (1 increase)	1.49	1.13–1.96	0.005	1.40	1.04–1.89	0.026
AF – yes	2.13	1.22–3.73	0.008	1.94	1.04–3.62	0.037
CPAP dropout – yes	4.67	2.50–8.73	<0.001	3.43	1.52–7.74	0.003
Unsuppressed SA – yes	2.64	1.48–4.74	0.001	2.30	1.21–4.38	0.011

AF, atrial fibrillation; BP, blood pressure; BNP, brain natriuretic peptide; CI, confidence interval; HR, hazard ratio; REM, rapid eye movement.

## Discussion

In this study, we demonstrated that the unsuppressed SA, residual AHI ≥ 15 on CPAP, was associated with increased clinical outcomes in patients HF with SA including either OSA or CSA accompanied by the presence of AF and the dropout from the CPAP therapy.

Previous studies reported that SA is associated with negative health consequences in patients with HF (11–13). Recurrent episodes of apneas during sleep followed by arousals generate repetitive hypoxia-reoxygenation, increased intrathoracic negative pressure, and exaggerated sympathetic nervous activity (6), all of which contribute to an increased ventricular afterload. CPAP is effective to suppress apneas and hypopneas in HF patients with OSA and in a half of patients with CSA (6), improving cardiac function, and potentially leading to improvements in the prognosis in patients with HF. However, previous clinical trial examining the efficacy of CPAP therapy in patients with HF and CSA, revealed that CPAP therapy did not significantly reduce incident clinical outcomes including mortality, heart transplantation or hospitalization for cardiovascular events (6, 8, 12, 13). Interestingly, a *post hoc* analysis of that trial reported that the study population whose AHI was reduced below 15/h on CPAP had better prognoses with regard to heart transplantation-free survival rates compared to those whom AHI on CPAP therapy was 15 or greater (14). Similar to this result, our study demonstrated that residual AHI ≥ 15 on CPAP therapy was associated with worse clinical outcomes in patients HF with SA, compared to those with residual AHI < 15. However, our study provides novel findings that such unsuppressed SA can be one of the important prognostic factor even in patients with HF and SA. Our subgroup analyses for type of SA (obstructive- or central-dominant SA) showed that unsuppressed SA had worse prognoses than suppressed SA in the obstructive-dominant SA while no statistical difference was observed between the two groups in the central-dominant SA. Type of SA had statistically significant interaction between clinical events and residual SA (*p* for interaction = 0.018). The finding is consistent with previous studies showing evidence on the detrimental effect of central apneas in patients with HF regardless of LVEF (15, 16).

Backdrop of the worse clinical outcomes in the unsuppressed- compared to the suppressed group, is partly explained by patients' characteristics in terms of relatively higher age, lower diastolic BP, significantly higher PNE level, and prevalence of AF in the unsuppressed group than those in the suppressed group. Indeed, the multivariate Cox regression analyses demonstrated that unsuppressed SA and presence of AF were associated with worse clinical outcomes. Considering the severer SA with more CSA on the diagnostic sleep study, which are generally associated with pulmonary congestion (17), in the unsuppressed group, we speculated that unsuppressed SA is indicatives of unfavorable patients’ backgrounds such as more volume overloaded and pulmonary congestion in association with more impaired cardiac function, all of which are associated with poor prognoses in patients with HF.

Patients with unsuppressed SA have greater total AHI with more hypoxic burden compared with those with suppressed SA. Recently, hypoxic burden rather than frequency of respiratory events (i.e., AHI) is regarded as having more impact of clinical outcome in patients with HF and SA (18). In the present study however, neither %TST SO_2 _< 90% nor lowest SO_2_ were not associated with increased risk of clinical events even in the univariate analyses. Relationships between hypoxic burden in patients with unsuppressed SA should be elucidated in the further study.

### Clinical implications and future perspectives

Our findings imply the needs of assessment for unsuppressed SA in patients with HF and SA treated with CPAP therapy. To stratify patients at risk of mortality and hospitalization, it should be enhanced to evaluate residual AHI especially in HF patients who have already been initiated into CPAP therapy. In such cases, because undergoing polysomnography may not be feasible, residual AHI which was provided by CPAP device will also be applicable because the AHI provided by CPAP or other positive airway pressure device is accurate and acceptable (19–21). We previously reported that in patients with unsuppressed SA, replacement of CPAP by bi-level positive airway pressure or adaptive-servo ventilation (ASV) led improvements of cardiac function with sufficient suppression of their SA (22, 23). However, there are no data suggesting the long-term prognostic impact of such sufficient suppression of SA in patients with HF and unsuppressed SA. ASV which is reported as providing short-term improvement of cardiac function in patients with HF and SA especially in cases with predominant CSA, failed to show long-term mortality benefit rather showed potentials to be harmful in patients with HF and predominant CSA in a large-scale clinical trial, the SERVE-HF trial (24). Another large-scale randomized controlled trial (25) examined the efficacy of ASV in patients with HF and SA including either OSA or CSA. In the trial, although ASV markedly reduced AHI, again ASV failed to show the benefit in the primary endpoint, which was the combination of cardiovascular hospitalizations, death from any cause, new-onset atrial fibrillation, and delivery of an appropriate discharge from an implantable cardioverter-defibrillator (26) in either overall, OSA subgroup or CSA subgroup. However, in CSA subgroup which did not include planned number of subjects because of the SERVE-HF trial leading to underpower to show the statistical significance, sufficient suppression of SA by ASV tended to have mortality benefit. In addition, although ASV is acceptable for HF patients with OSA with some CSA component, whether ASV provide beneficial effects on clinical outcomes in patients with almost pure OSA and no/limited CSA component remains controversial. Further studies are needed to examine how we treat residual AHI in those patients to improve their prognoses.

### Limitations

This was retrospective analyses of the single-center observational study with a relatively small sample size. Moreover, since the present study was observational in nature, other unknown confounders might have affected the prognosis even after the multivariate analysis. Therefore, our data should be interpreted carefully, and further studies with larger sample size and intervention to improve unsuppressed SA are required to confirm our data. A recent study suggested that variability of the residual AHI may alter particularly in patients with HF (27). Thus, status regarding unsuppressed SA may also alter. Since our data were collected between 2001 and 2005, during which contemporary clinical practice of HF were not yet established, pharmacological therapy for HF were not fully optimized, which might have affected the observed outcomes in the study population.

## Conclusions

Our study demonstrated that patients with HF and SA were associated with worse prognosis in patients with residual AHI ≥ 15 compared to those with residual AHI < 15.

## Data Availability

The raw data supporting the conclusions of this article will be made available by the authors, without undue reservation.
